# Editorial for Special Issue “Advances in the Pathogenesis and Treatment of Immune-Mediated Inflammatory Diseases”

**DOI:** 10.3390/ijms23158415

**Published:** 2022-07-29

**Authors:** Jan Piet van Hamburg, Sander W. Tas

**Affiliations:** 1 Department of Rheumatology and Clinical Immunology, Amsterdam Rheumatology and Immunology Center, Amsterdam University Medical Centers, University of Amsterdam, Meibergdreef 9, 1105 AZ Amsterdam, The Netherlands; j.p.vanhamburg@amsterdamumc.nl; 2 Department of Experimental Immunology, Amsterdam University Medical Centers, University of Amsterdam, Meibergdreef 9, 1105 AZ Amsterdam, The Netherlands

This Special Issue focuses on the rapidly evolving field of immune-mediated inflammatory diseases (IMIDs) and the achievements that were made over the last 10 years. With the advent of novel technologies such as single-cell RNA sequencing (scRNAseq) and state-of-the-art imaging techniques, researchers in our field have made tremendous progress in unraveling the pathogenesis of autoimmune diseases such as rheumatoid arthritis (RA), vasculitis and systemic lupus erythematosus, as well as “autoinflammatory” diseases such as spondyloarthritis (SpA) and fever syndromes. The novel technologies have also provided insights into the contribution of specific (subsets of) immune cells and stromal cells to the inflammatory response, and into the molecular pathways associated with the pathological processes involved. These advances have led to the identification of promising new therapeutic targets for these diseases and paved the way for novel treatments. However, for rare IMIDs, we are far from fully understanding the mechanisms leading to the development and progression of disease, as well as the response to therapy. A better understanding of the molecular and cellular signatures of these diseases may elucidate potential new treatment options.

Several contributions to this issue focus on the role of B lineage cells in systemic autoimmune diseases. Merino Vico et al. review the current literature and highlight the importance of these cells in ANCA-associated vasculitis [1]. In an original article, Neys et al. investigated potential alterations in BAFF receptor expression and BCR signaling in B cells of patients with primary Sjogren’s syndrome (pSS) at diagnosis [2]. Using (phospho-)flow cytometry, the phosphorylation of BCR signaling molecules and BAFF receptor (BAFFR) expression was quantified in circulating B cell subsets of pSS patients compared to non-pSS sicca and healthy controls (HCs). Interestingly, BCR signaling activity was comparable in these groups, whereas BAFFR expression was significantly downregulated in pSS patients, correlating with pSS-associated alterations in B cell subsets. This suggests that reduced BAFFR expression could be an early sign of B cell involvement and a diagnostic marker for pSS. B cells not only play an important role in autoimmune diseases, but are likely also crucial in chronic inflammatory diseases that have long been considered autoinflammatory, including SpA. The mounting evidence for this in the recent literature is reviewed by Wilbrink et al. who clearly show that B lineage cells are involved in the pathogenesis of ankylosing spondylitis, which is illustrated by clear alterations in circulating B cell populations and the formation of (autoreactive) antibodies, along with tertiary lymphoid structures (TLO) with B cell infiltrates at sites of inflammation [3]. In an experimental study, Jeucken et al. investigated the impact of transmembrane (tm) TNF overexpression on secondary lymphoid organ (SLO) development and function in a mouse model (tmTNFtg mice) [4]. Using advanced 3D ultramicroscopy, striking alterations in peripheral lymph nodes and spleen were identified that would not have been uncovered with conventional microscopy methods, demonstrating that tightly regulated tmTNF is important for proper SLO development and function. Anang et al. review the role of (ectopic) germinal centers in the process of autoantibody generation in SLO and TLO in RA and discuss (pre-)clinical approaches to target various germinal center-associated processes as a novel therapeutic strategy [5]. The last manuscript in this sequence of papers on SLO and TLO addresses the key role of gut-associated lymphoid tissue in the pathogenesis of inflammatory bowel diseases (IBD) [6].

The importance of a tightly regulated innate immune system is underscored by an intriguing report demonstrating that efficient neutrophil activation requires the simultaneous ligation of two different receptors by two independent stimuli [7]. The requirement for the stimulation of several distinct receptors is consistent with the potentially detrimental impact of neutrophil activation on the host and the multiple levels of regulation that govern activation. Similarly, an interesting study by Geyer et al. shows that C-reactive protein (CRP), an acute-phase protein in humans that is produced in high quantities during infection and inflammatory conditions, is not only a marker of inflammation, but also a direct mediator of inflammation by eliciting IL-23 production by monocytes, which may contribute to shaping immune responses during infections and/or chronic inflammatory diseases [8]. The IL-23/IL-17 axis plays a critical role in various IMIDs, such as SpA. Hammoura et al. showed that dual blockade of TNF and IL-17 in the HLA-B27/human β2-microglobulin transgenic rat model for SpA significantly reduces peripheral arthritis and spondylitis, including new bone formation [9].

Although the involvement of immune cells in the pathogenesis of IMIDs has long been recognized, the crucial role of stromal cells in these diseases has only recently been appreciated. The importance of stromal cells was recently highlighted by an innovative approach in which pathogenic cells were depleted by photodynamic therapy (tPDT) that activated photosensitizer (IRDye700DX)-conjugated antibodies directed against fibroblast activation protein (FAP) on fibroblasts. This novel phototherapy strategy led to the dose-dependent depletion of primary skin fibroblasts revealed in lesional skin biopsies of systemic sclerosis (SSc) patients upon light exposure in a 3D culture system [10]. In another study, the potential of targeting the intracellular TGF β-activated kinase 1 (TAK1) in human RA synovial fibroblasts with a small-molecule inhibitor was investigated in vitro, demonstrating the inhibition of inflammatory responses in these cells by targeting multiple signal transduction pathways, most notably JAK/STAT signaling [11]. In an exhaustive review article, den Braanker et al. discuss the recent insights generated via scRNAseq regarding the heterogeneity and functional diversity of lymphatic endothelial cells in health and disease, which underscores this technology’s potential to advance our knowledge on the plethora of cells involved in the pathogenesis of IMIDs [12].

Besides these more basic and translational studies, the Special Issue includes comprehensive review articles on the pathophysiology and treatment of idiopathic inflammatory myopathies [13] and the role of the immune system in IBD-associated tumorigenesis [14].

Collectively, the articles in this Special Issue illustrate the breadth of recent advances in IMIDs research and demonstrate the potential of novel technologies such as advanced microscopy methods and (sc)RNAseq to dissect pathological processes and identify novel therapeutic targets to solve unmet clinical needs in these diseases. The future is looking bright!
